# Screening a Strain of *Klebsiella* sp. O852 and the Optimization of Fermentation Conditions for *Trans*-Dihydrocarvone Production

**DOI:** 10.3390/molecules26092432

**Published:** 2021-04-22

**Authors:** Li Chen, Lu-Lu Zhang, Jing-Nan Ren, Xiao Li, Gang Fan, Si-Yi Pan

**Affiliations:** 1 Key Laboratory of Environment Correlative Dietology, Ministry of Education, College of Food Science and Technology, Huazhong Agricultural University, Wuhan 430070, China; chenli0915@webmail.hzau.edu.cn (L.C.); renjingnan@mail.hzau.edu.cn (J.-N.R.); lixiao1@webmail.hzau.edu.cn (X.L.); 2 College of Food Science and Technology, Henan University of Technology, Zhengzhou 450001, China; zhanglulu@webmail.hzau.edu.cn

**Keywords:** *Klebsiella* sp. O852, limonene, biotransformation, *trans*-dihydrocarvone, optimization

## Abstract

Flavors and fragrances have high commercial value in the food, cosmetic, chemical and pharmaceutical industries. It is interesting to investigate the isolation and characterization of new microorganisms with the ability to produce flavor compounds. In this study, a new strain of *Klebsiella* sp. O852 (accession number CCTCC M2020509) was isolated from decayed navel orange (*Citrus sinensis* (L.) Osbeck), which was proved to be capable of converting limonene to *trans*-dihydrocarvone. Besides, the optimization of various reaction parameters to enhance the *trans*-dihydrocarvone production in shake flask was performed for *Klebsiella* sp. O852. The results showed that the yield of *trans*-dihydrocarvone reached up to 1 058 mg/L when *Klebsiella* sp. O852 was incubated using LB-M medium for 4 h at 36 °C and 150 rpm, and the biotransformation process was monitored for 36 h after adding 1680 mg/L limonene/ethanol (final ethanol concentration of 0.8% (*v/v*)). The content of *trans*-dihydrocarvone increased 16 times after optimization. This study provided a basis and reference for producing *trans*-dihydrocarvone by biotransformation.

## 1. Introduction

Aromas and fragrances have high commercial value in the food, cosmetic, chemical, agricultural, tobacco, flavor and pharmaceutical industries. In 2017, the global market of aromas and fragrances reached US$ 28.2 billion. It is estimated to grow at 4.9 per cent per annum to reach US$ 36 billion in 2022 [1]. Thus, the prospect of the industrialization of the aromas and fragrances is very bright, and the production of flavor compounds using various approaches has attracted great attention. Currently, most flavor compounds are obtained by chemical synthesis [2], which have the disadvantages of a high energy consumption and pollution [3]. The products obtained through chemical synthesis cannot be considered as “natural” products [4]. With the increasing awareness of the link between diet and health, consumers prefer products labelled as “natural” or “bio-flavors” [5]. Biotransformation is a promising alternative method for the production of “natural” aromas. It has the advantages of mild reaction conditions, less by-products, environmental friendliness, high regioselectivity and enantioselectivity, and the generation of the transformation products can be labeled as “natural” [6]. Among the substrates used in the biotransformation process, terpenes could be highlighted based on their versatility and low cost, especially limonene.

Limonene is the major volatile constituent of citrus fruits, and it is natural, low cost, easily available, and can be used as a starter for the production of flavors [7]. The biotransformation of limonene is able to generate products with high added value, such as perillyl alcohol, carvone, carveol, dihydrocarvone, and α-terpineol [8]. The market values of these compounds are 10 to 30 times higher than that of limonene [2,9]. In addition, the oxygenated derivatives of limonene have multiple pharmacological activities including anti-microbial, anti-inflammatory [2], anti-oxidant, anti-proliferative [10], anti-cancer [11,12], and so on. These compounds have the effect of reducing pain and producing a sense of calm [13]. Therefore, the production of these flavor compounds is expected to have a further development in the coming years. An important step in this direction is the isolation of microorganisms, which can catalyze specific reaction and obtain new bio-flavor products at a relatively low cost.

Recently, many researches have reported that many strains (fungi, bacteria and yeasts) could yield aroma compounds using limonene as the precursor [9,14,15,16]. However, the limonene biotransformation process is still limited because of the high volatility and low solubility of this precursor, the low transformation rates, high cytotoxicity of both precursor and product, and so on [17]. Hence, we decided to isolate limonene-tolerant microorganisms from an extreme environment. We screened a strain that is both resistant to limonene and high-yielding, and it can tolerate cytotoxicity and increase the conversion rate and the content of aromatic compounds. Furthermore, considering the application of limonene and based on the references of solvent-tolerant strain screening [16,17,18], we isolated and screened the strains from the soil of the citrus processing plant, citrus fruits and oilfield. Citrus fruits are abundant with limonene where it is believed to have strains more adapted to the limonene-containing environment [7].

The objective of this study is to screen aroma-producing microorganisms and optimize the fermentation parameters of the selected strain in order to enhance the production of the aroma compounds. This study describes the strain *Klebsiella* sp. O852 screened from the decayed navel orange, which can utilize limonene as a sole carbon source and have the potential to yield *trans*-dihydrocarvone. There are only a few reports describing the production of *trans*-dihydrocarvone by *Klebsiella* sp. *Klebsiella* sp. is gram negative and belongs to the family of *Enterobacteriaceae* [19]. *Klebsiella* sp. has a potential value of application because of its good environment adaptation ability, fast growth, high conversion rate, short fermentation period and broad substrate specificity [20,21]. *Klebsiella* sp. O852 is able to use limonene to produce aroma compounds for the food and cosmetic industry. It is of great interest to avoid waste pollution caused by the citrus processing residues, and to improve the utilization of by-products in citrus processing.

## 2. Results and Discussion

### 2.1. Isolation and Screening of Limonene-Resistant Microorganisms

**Figure 1:**
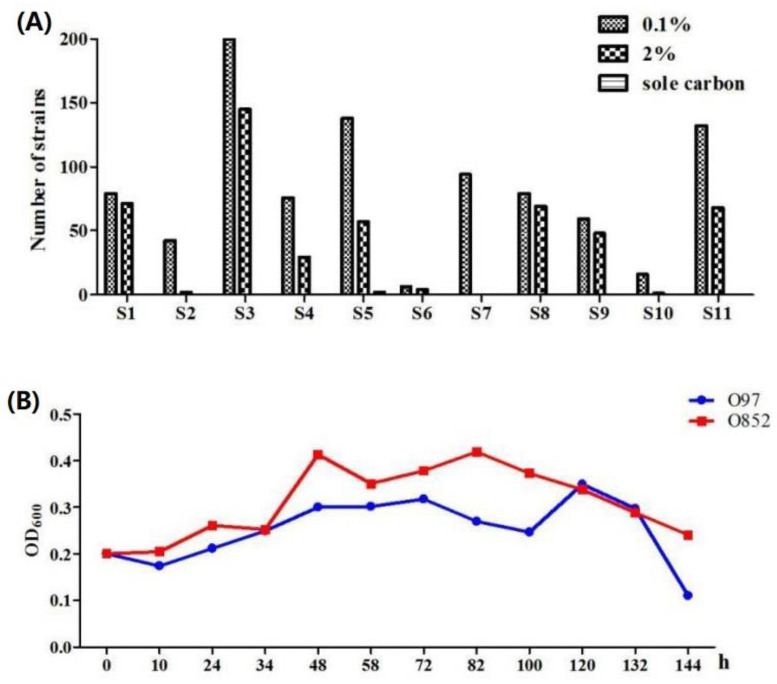
Isolation and screening of limonene-resistant microorganisms. (**A**) Microbial isolate resistance screening results of 11 samples with limonene. (**B**) Effect of 1% limonene on the growth of strain O97 and strain O852 in M9 medium. S1, soil of citrus processing plant; S2, soil of citrus fruits; S3, soil of Jianghan Oilfield; S4, peel of fresh Gannan navel orange; S5, peel of rotten Gannan navel orange; S6, peel of fresh Anyue lemon; S7, peel of rotten Anyue lemon; S8, peel of fresh Guanxi pomelo; S9, peel of rotten Guanxi pomelo; S10, peel of fresh Satsuma mandarin; S11, peel of rotten Satsuma mandarin.

As shown in Figure 1A, 921 microorganisms in total were isolated in yeast and malt extract (YM) medium containing 0.1% limonene. Among them, 494 microorganisms were grown in YM medium containing 2% limonene and presented a satisfactory growth. Notably, most isolated fungi were not able to grow in YM medium containing 2% limonene. Limonene and monocyclic monoterpene could be dissolved in fungal membrane due to its hydrophobicity. This led to the increase in membranes fluidity, the loss of membrane integrity, and the increase of membrane permeability [22]. Thus, the fungi might be more susceptible to limonene compared to bacteria and yeast. Finally, 2 strains (O97 and O852) were able to grow in M9 medium containing limonene as the sole carbon source, indicating the existence of a limonene-degrading pathway. This suggested that strain O97 and strain O852 could be able to transform limonene to produce an oxygenated compound. The effects of 1% limonene on the growth of strain O97 and strain O852 in M9 medium were presented in Figure 1B.

### 2.2. Phylogenetic Identification of the Isolates

**Figure 2:**
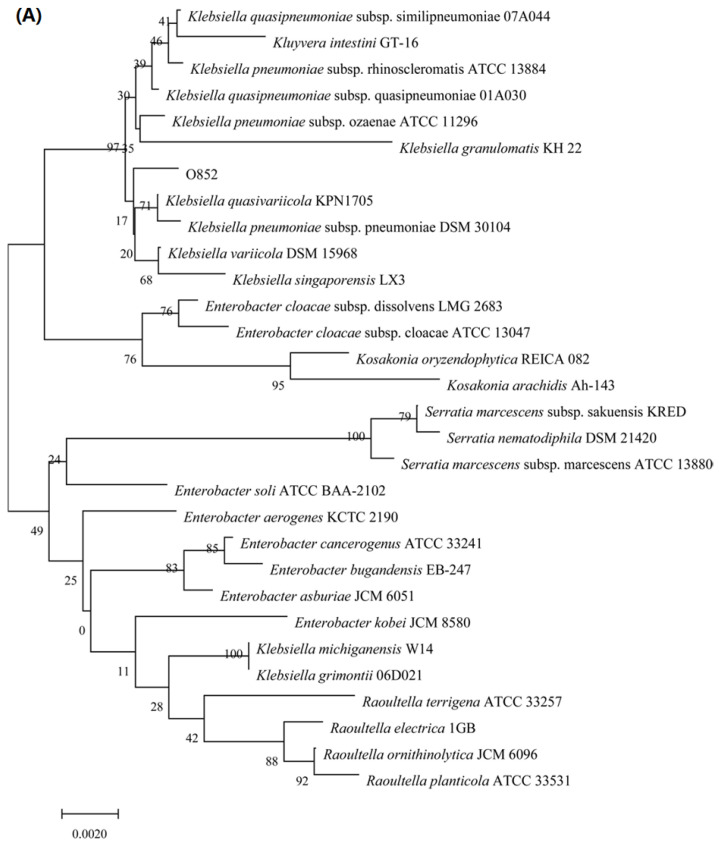

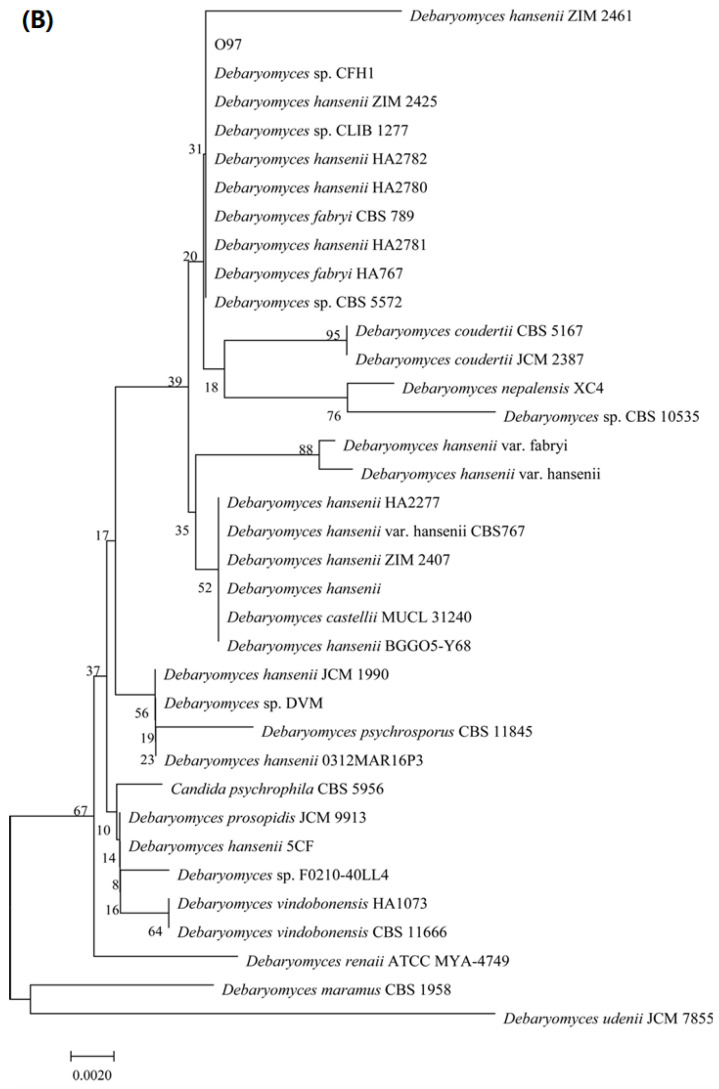
Identification of limonene resistant microorganisms. (**A**) Phylogenetic tree of strain O852 based on the 16S rDNA gene sequence. (**B**) Phylogenetic tree of strain O97 based on the ITS gene sequence.

The 16S rDNA and ITS sequencing methods were used to characterize these two strains. The phylogenetic trees of isolates O97 and O852 were constructed. As shown in Figure 2A, the 16S rDNA gene sequences of strain O852 had 99% similarity to >100 species in the genus *Klebsiella*. This similarity suggested that strain O852 belonged to this genus. Therefore, this strain was named as *Klebsiella* sp. O852 and the accession numbers was MW757187 from GenBank. Moreover, *Klebsiella* sp. O852 was deposited at the China Center for Type Culture Collection (CCTCC, Wuhan, China) with the accession number CCTCC M2020509.

Meanwhile, the ITS sequences of strain O97 had 99% similarity to >100 species in the genus *Debaryomyces*, suggesting that strain O97 belonged to this genus (Figure 2B). This strain was named as *Debaryomyces* sp. O97. The ITS sequences of *Debaryomyces* sp. O97 were submitted to GenBank and the accession number MW757188 was given.

### 2.3. Limonene Biotransformation

*Klebsiella* sp. O852 and *Debaryomyces* sp. O97 were selected to conduct the biotransformation experiments due to their capacity of using limonene as the sole carbon source in a mineral medium. It can be observed in Table 1 that a mixture of products (*trans*-dihydrocarvone, trace amounts of γ-terpinene, p-menth-1-en-9-al, carvone, as well as a relatively high amount of 1,2-limonene epoxide) were obtained by *Klebsiella* sp. O852. Among them, *trans*-dihydrocarvone was the main biotransformation product. At different biotransformation times (24 h, 48 h and 72 h), there was a significant difference (*p* < 0.05) between the content of *trans*-dihydrocarvone and other bioconversion products. The content of *trans*-dihydrocarvone reached the maximum of about 62.86 ± 5.95 mg/L at 24 h. Subsequently, the *trans*-dihydrocarvone content decreased with the increasing biotransformation time (48–72 h), which might be explained by the depletion and lack of nutrients [23]. Besides, the production of other by-products may also lead to the decrease in the *trans*-dihydrocarvone yield. In the study of Li et al. (2020), by-products were generated with the increase in biotransformation time, resulting in a decline in product yield [24]. Furthermore, with the increase in biotransformation time, the microorganism gradually entered the stationary phase and the decline phase, leading to a decrease in the catalytic capacity.

On the other hand, carvone, 1,2-limonene epoxide, *trans*-carveol, γ-terpinene, along with trace amounts of perillyl alcohol, were found as the products by *Debaryomyces* sp. O97. The carvone was the main biotransformation product of *Debaryomyces* sp. O97 and its content reached the maximum of about 64.03 ± 22.09 mg/L at 48 h. However, it was not found at 24 h and 72 h.

*Klebsiella* sp. was mainly distributed in the respiratory and intestinal tract of healthy humans and animals. It was a conditional pathogenic bacteria which can cause the disease only in exceptional circumstances [25]. Under normal conditions, *Klebsiella* sp. is usually not harmful for humans and animals. It has the ability to utilize glucose and glycerol to produce chemicals [20]. For example, *Klebsiella oxytoca* was capable of converting glucose to generate 2,3-butanediol [26]. *Klebsiella pneumoniae* can utilize glycerol as a carbon source and catalyze glycerol to yield 1,3-propanediol [27]. When glycerol was used as the sole carbon source, *Klebsiella* sp. AA405 can produce 1,3-propanediol, 2,3-butanediol and 3-hydroxypropionic acid [20]. Additionally, Zhou et al. (2017) found that *Klebsiella* sp. could produce secoisolariciresinol by biotransformation of precursors in defatted flaxseeds [25]. Hence, *Klebsiella* sp. was a promising industrial strain.

Based on our findings, *Klebsiella* sp. O852 may have potential applications in limonene biotransformation and production of bio-flavor compounds. It can be noted from Table 1 that the content of *trans*-dihydrocarvone was the highest in all product of *Klebsiella* sp. O852. The oxygenated monoterpene *trans*-dihydrocarvone with a spearmint-like odor was a component of the dill oil, caraway seeds, the essential oils of *Mentha* spicate [28]. It was formed as a mixture of two isomers: (1*R*, 4*R*)-(+)-dihydrocarvone and (1*S*, 4*R*)-(+)-dihydrocarvone. Dihydrocarvone can be used not only as a flavoring additive in the food industry, but also as a chiral building block for the synthesis of different molecules of biological interest, such as keto decalin derivatives, tetraoxane derivatives, terpenes thujopsene and (+)-decipienin A [29,30]. Besides, dihydrocarvone had an inhibitory effect on bacterial and fungal growth, and presented good performance as an insect repellent [31].

**Figure 3:**
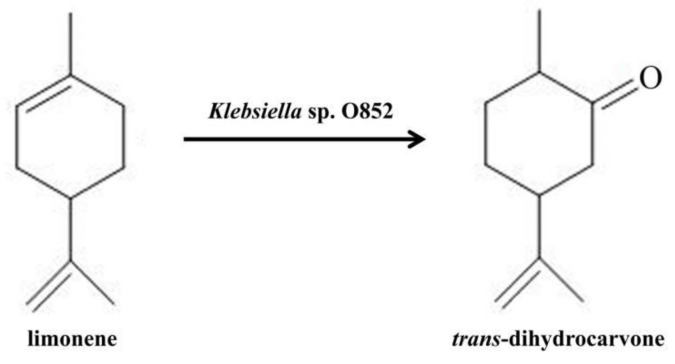
Bioconversion of limonene to *trans*-dihydrocarvone using *Klebsiella* sp. O852.

Taken together, *Klebsiella* sp. O852 was further studied as a potential strain for the production of *trans*-dihydrocarvone (Figure 3).

### 2.4. Optimization of Trans-Dihydrocarvone Production by Klebsiella sp. O852 Using Single Factor Design

To obtain a better performance of the biotransformation, a series of factors influencing the biotransformation conditions of limonene to *trans*-dihydrocarvone by *Klebsiella* sp. O852 were explored.

#### 2.4.1. Growth Curve of *Klebsiella* sp. O852

**Figure 4:**
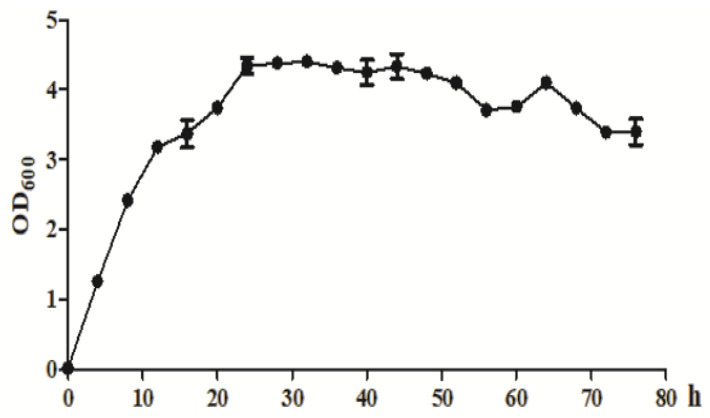
The growth curve of *Klebsiella* sp. O852 (cultivation conditions were at 30 °C and 150 rpm).

As shown in Figure 4, *Klebsiella* sp. O852 entered the logarithmic growth phase after 4 h of inoculation and entered the stationary phase after 24 h, which was followed by the senescent phase after 52 h.

#### 2.4.2. Effect of Metal ion on the Production of Trans-Dihydrocarvone by *Klebsiella* sp. O852

**Figure 5:**
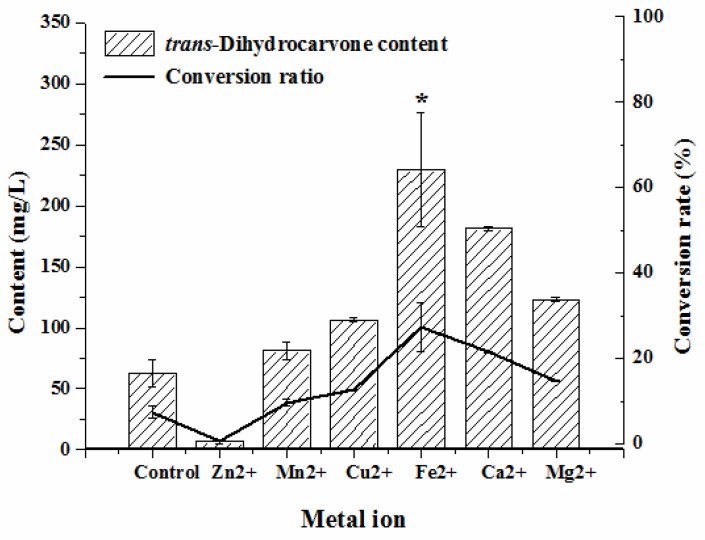
Effect of metal ions on the production of *trans*-dihydrocarvone by *Klebsiella* sp. O852 (cultivation conditions were at 30 °C and 150 rpm, limonene of 840 mg/L was added as substrate) * *p* < 0.05 vs. control group.

Apparently, all metal ions could positively affect the biotransformation except for Zn^2+^ (Figure 5). The addition of Zn^2+^ reduced the *trans*-dihydrocarvone content compared to the control group. This might be because the enzyme involved in this biotransformation was inhibited by Zn^2+^. Both the *trans*-dihydrocarvone concentration and conversion ratio were the highest after adding Fe^2+^ (*p* < 0.05). A previous study noted that limonene-6-hydroxylase (CYP71D18) was responsible for the hydroxylation of limonene to carveol [32]. CYP450 were a superfamily of heme (iron protoporphyrin IX)-containing monooxygenase enzymes [33], so the activity of CYP450 may be enhanced by adding Fe^2+^. Thus, Fe^2+^ should be added to the medium to obtain the best biotransformation effect.

#### 2.4.3. Effect of Biotransformation Conditions on the Production of Trans-Dihydrocarvone by *Klebsiella* sp. O852

**Figure 6:**
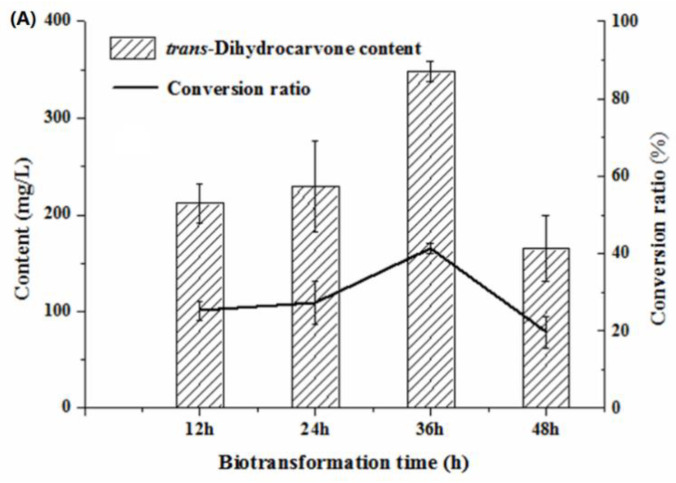

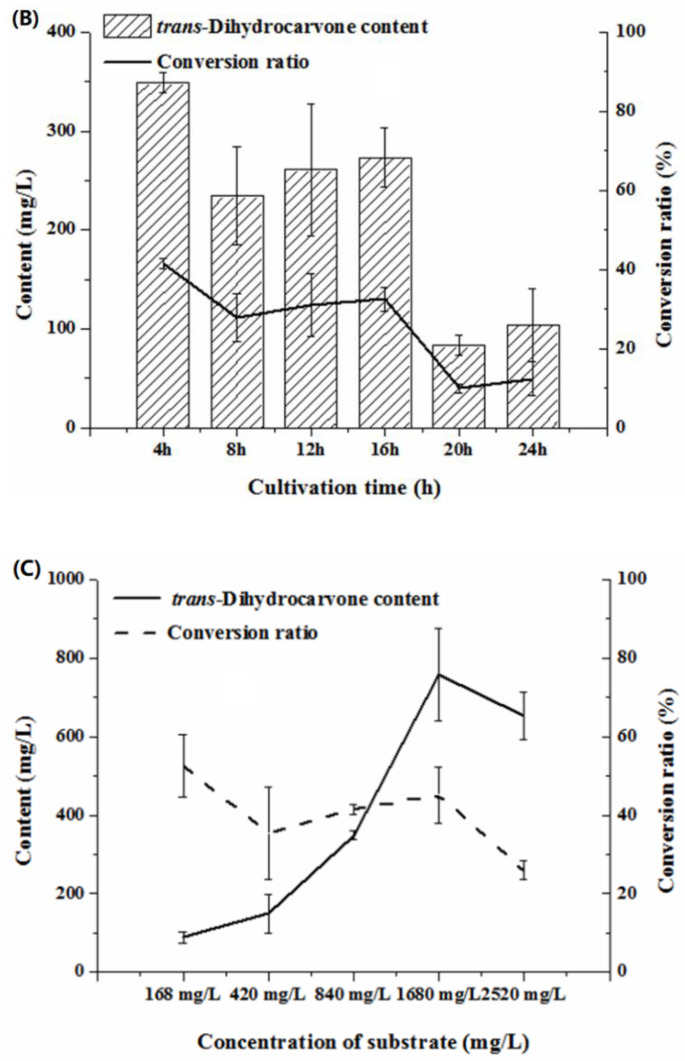
Effect of biotransformation conditions on the production of *trans*-dihydrocarvone by *Klebsiella* sp. O852 (cultivation conditions were at 30 °C and 150 rpm). (**A**) Effect of biotransformation time on the production of *trans*-dihydrocarvone. (**B**) Effect of cultivation time on the production of *trans*-dihydrocarvone. (**C**) Effect of substrate concentration on the production of *trans*-dihydrocarvone.

In general, the yield of the products was low at the shorter biotransformation time [34]. It can be seen that *trans*-dihydrocarvone content increased first and then decreased with the increasing biotransformation time. The *trans*-dihydrocarvone concentration reached the maximum value when the biotransformation time was 36 h (Figure 6A). After 36 h of the biotransformation, the *trans*-dihydrocarvone concentration decreased because of the depletion of nutrients and production of other by-products in the culture medium [35]. This result was consistent with the result of limonene biotransformation experiments. It indicated that the *trans*-dihydrocarvone yield decreased with the increase of biotransformation time. Therefore, 36 h was considered as the optimum biotransformation time for the production of *trans*-dihydrocarvone.

The cultivation time was one of the most important factors in biomass production that could affect the subsequent biotransformation process and the yield of the products [35]. In this study, it was observed that *trans*-dihydrocarvone was produced at all growth phases, but the amounts of the product were different. The yield of the product decreased with the increase in cultivation time, and the maximum *trans*-dihydrocarvone content was achieved when the cultivation time was 4 h (logarithmic growth phase) (Figure 6B). It was suggested that 4 h was enough to accumulate biomass and promote biotransformation. Hence, the optimum cultivation time was 4 h. The longer cultivation time could lead to cells hypoxia, which reduced the *trans*-dihydrocarvone yield [36]. Li et al. (2020) found that the yield of the product reached the highest level when the substrate was added in the logarithmic growth phase [24]. Similar results were also reported by Gloria et al. (2011) [37]

The high cytotoxicity and high volatility of both substrate and product must be taken into consideration in the biotransformation process. It was clearly showed that the content of *trans*-dihydrocarvone increased first and then decreased with the increase in substrate concentrations (168–2520 mg/L), and it reached the maximum value when the limonene concentration was 1680 mg/L (Figure 6C). A previous study found that the maximal concentration of α-terpineol was obtained after adding 840 mg/L limonene using the *Penicillium digitatum* DSM 62,840 [38]. The use of a high concentration of limonene also presented higher concentrations of product formation, possibly because bacteria seem to be more insensitive to limonene compared to fungi [39]. It was maybe one of the reasons that no fungi strains were isolated in this study. This result was similar to the other study, which optimized the bioconversion of carvone to dihydrocarvone using the filamentous fungus *Phoma* sp. It was observed that higher conversion yields were obtained using lower amounts of substrate [40]. Moreover, the best conversion ratio was obtained after adding 168 mg/L limonene, but the product concentration was low. Considering the product concentration and conversion ratio, 1680 mg/L was chosen as the optimal substrate concentration.

#### 2.4.4. Effect of Co-Solvent on the Production of Trans-Dihydrocarvone by *Klebsiella* sp. O852

**Figure 7:**
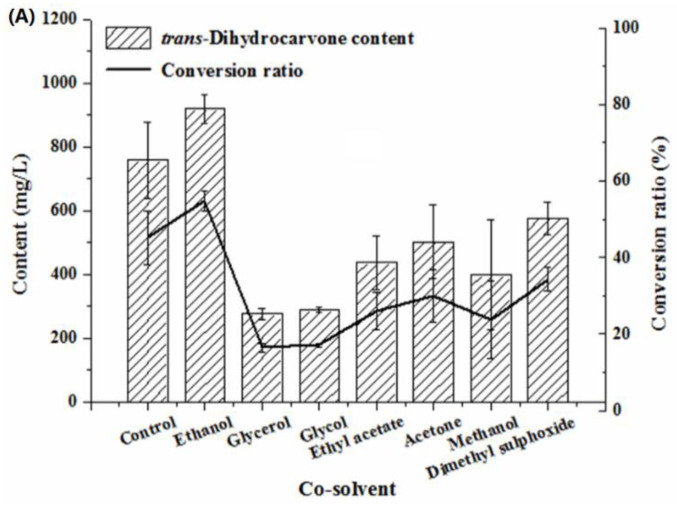

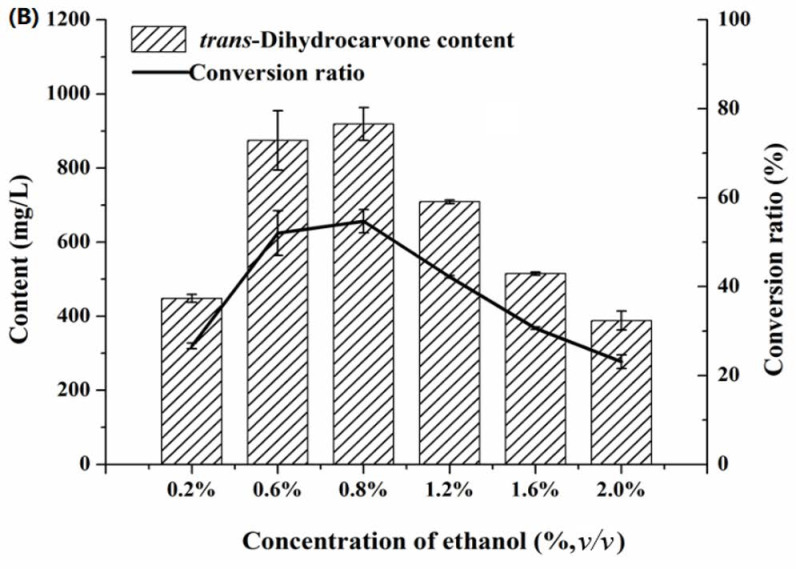
Effect of co-solvent on the production of *trans*-dihydrocarvone by *Klebsiella* sp. O852 (cultivation conditions were at 30 °C and 150 rpm, limonene of 1680 mg/L was added as substrate) (**A**) Effect of different co-solvent on the production of *trans*-dihydrocarvone. (**B**) Effect of ethanol concentration on the production of *trans*-dihydrocarvone.

Generally, organic solvents were supplemented to biotransformation processes to increase substrate solubility. However, this may have a negative impact on enzyme activity [41]. As shown in Figure 7A, the biotransformation was inhibited by glycerol, glycol, ethyl acetate, acetone, methanol and dimethyl sulfoxide. This fact may be attributed to a toxic effect caused by these solvents on the biomass of strain O852 [42]. The addition of ethanol had a considerable positive effect on the formation of *trans*-dihydrocarvone, which was indicated by an increase in the conversion ratio from 45% up to 55%. Therefore, ethanol was chosen as the co-solvent for the biotransformation. Tai et al. (2016) reported that the best yield of product was achieved using 0.4% ethanol as co-solvent, and biotransformation was promoted by methanol, acetone, dimethyl sulfoxide and ethyl acetate [38].

It can be observed that there was a positive effect on the bioconversion when ethanol was selected as co-solvent. Therefore, the biotransformation was conducted by adding 0.2%, 0.6%, 0.8%, 1.2%, 1.6% and 2.0% (*v/v*) ethanol. The result was represented in Figure 7B. The biotransformation was affected by the concentration of ethanol. The *trans*-dihydrocarvone content increased first and then decreased with the increase of ethanol concentration, and it reached the maximum value when the concentration of ethanol was 0.8% (*v/v*).

#### 2.4.5. Effect of Culture Conditions on the Production of Trans-Dihydrocarvone by *Klebsiella* sp. O852

**Figure 8:**
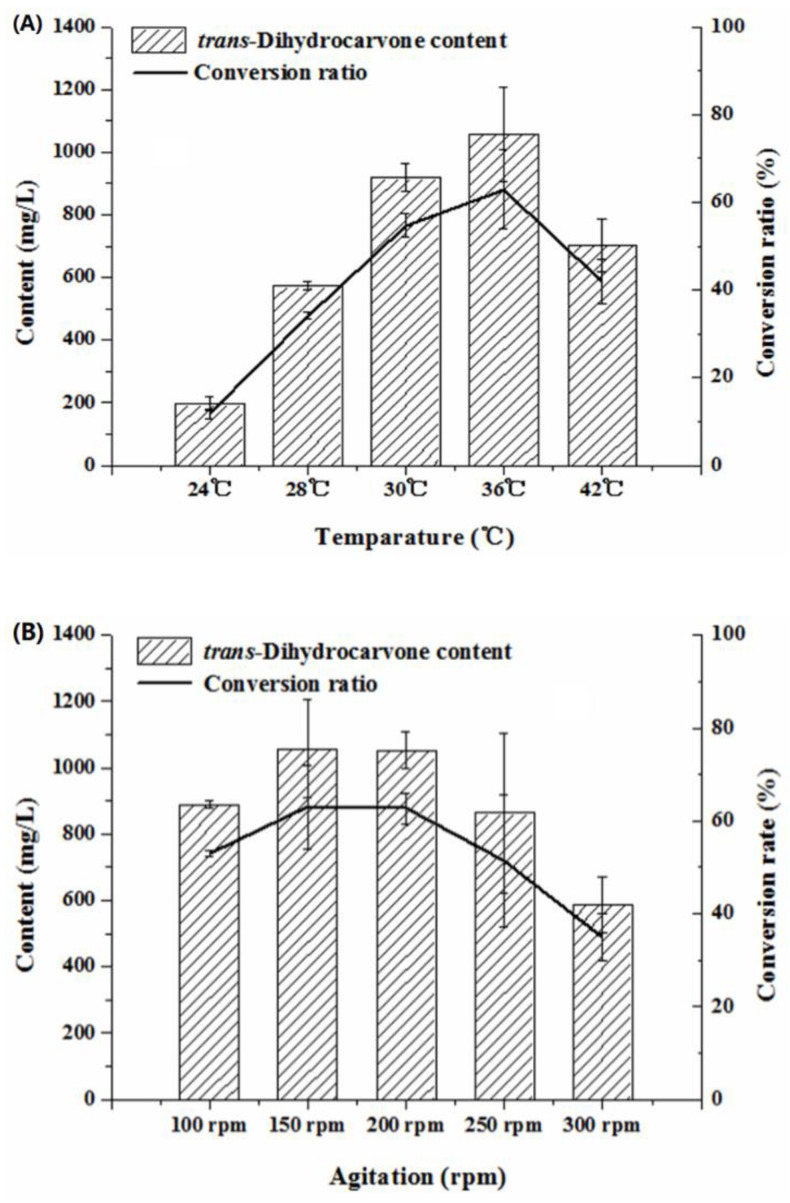
Effect of culture conditions on the production of *trans*-dihydrocarvone by *Klebsiella* sp. O852 (limonene of 1680 mg/L was added as substrate). (**A**) Effect of temperature on the production of *trans*-dihydrocarvone. (**B**) Effect of agitation on the production of *trans*-dihydrocarvone.

Temperature was an important factor for microbial growth and product yields by influencing enzyme activity, enzyme stability, substrate solubility and nutrient intake during the biotransformation process [43]. High or low temperatures will be harmful to the enzymatic systems of the bacteria, thereby affecting the biotransformation process [44]. Low temperature may lead to a slow rate of reaction, and high temperature may result in the aggregation of protein and the inactivation of the enzyme [35]. In this study, the *trans*-dihydrocarvone content increased first and then decreased with the increase of the temperature (24–42 °C) (Figure 8A). The *trans*-dihydrocarvone concentration reached the maximum value when the temperature was 36 °C. Hence, 36 °C was used in the following experiments. A similar study was reported by Parate et al. (2018) who optimized the bioconversion of glycerol to 2,3-butanediol using *Klebsiella pneumoniae*. The results showed that the 2,3-butanediol content increased first and then decreased with the increase of the temperature (30–60 °C) and reached the highest value at 37 °C [45].

On the other hand, the agitation also directly affected the bioconversion. The agitation could control the dissolved oxygen in the fermentation liquid. Low agitation could decrease the dissolved oxygen in the medium, which made it difficult to satisfy the strain needs for the oxygen. High agitation could increase the mechanical damage on the cell, which directly affected the growth of the cells [46]. The *trans*-dihydrocarvone content increased first and then decreased with the increase of agitation speed (100–300 rpm) in this study (Figure 8B). When the agitation was 150 rpm, the *trans*-dihydrocarvone content and conversion ratio reached a maximum of about 1 058 mg/L and 63%, respectively. Taken together, the content of *trans*-dihydrocarvone increased 16 times after the optimization. These results suggested that the optimization of catalytic conditions can obviously increase the yield of *trans*-dihydrocarvone by *Klebsiella* sp. O852.

In general, dihydrocarvone may be obtained either by carvone hydrogenation or by limonene oxidation to the best of our knowledge. Most research has focused on carvone hydrogenation [29,47,48], while research on limonene oxidation are still insufficient. Some research has reported that 80–90% yield of dihydrocarvone could be produced by the transformation of limonene oxide in 1,4-dioxane solutions using heteropoly acid as catalyst, or in dialkylcarbonates solutions using silica-supported tungstophosphoric acid as a catalyst [49,50]. However, dihydrocarvone obtained using this method (chemical synthesis) had a low quality and cannot be labeled as “natural”. Besides, environmental liabilities (use of large volumes of organic solvents) were generated by chemical synthesis [2].

In most studies, dihydrocarvone obtained using biotransformation was to use carvone rather than limonene as a substrate. Ghasemi et al. (2009) showed that *Oocystis pusilla* can convert limonene to *trans*-carveol and carvone, convert carvone to *trans*-dihydrocarvone and *cis*-dihydrocarvone, and convert carveol to carvone and *trans*-dihydrocarvone [15]. Mahler et al. (2019) performed overexpression of ene reductase from *Nostoc* sp. PCC7120 in *Escherichia coli* and proved that these *Escherichia coli* whole-cell biocatalysts could catalyze (*R*)-carvone to yield (2*R*,5*R*)-dihydrocarvone [29]. Furthermore, dihydrocarvone was also generated using carvone as a substrate by *Spodoptera litura* [48] and the enzymatic system of vegetables [47]. In this study, natural and high quality dihydrocarvone was produced using limonene as a substrate by *Klebsiella* sp. O852. Limonene, by-product of the citrus industry, had a lower cost and was a good source to obtain *trans*-dihydrocarvone compared to carvone or limonene oxide, and therefore achieves higher yields. Moreover, conversion of limonene to dihydrocarvone can occur via a three-step reaction, with carveol and carvone as the intermediate product. Limonene was first converted into carveol, followed by the reduction of carveol to carvone, which was then converted into dihydrocarvone [51]. In this study, fewer by-products and intermediate products (carveol and carvone) were produced during the biotransformation process. On the other hand, *Klebsiella* sp. O852 that utilized limonene as a sole carbon source could be highly tolerant to limonene and transform limonene into *trans*-dihydrocarvone with high selectivity and high yield. It was demonstrated that *Klebsiella* sp. O852 was an excellent biocatalyst for *trans*-dihydrocarvone synthesis. This study described an efficient, rapid, inexpensive and feasible biotransformation system for *trans*-dihydrocarvone production.

## 3. Materials and Methods

### 3.1. Materials

*R*-(+)-limonene (97%) was obtained from Tokyo Chemical Industry Co., Ltd. (Tokyo, Japan). (+)-Dihydrocarvone was purchased from Beijing Innochem Science and Technology Co. Ltd. (Beijing, China). The carvone and perillyl alcohol were purchased from Shanghai Yuanye Bio-Technology Co., Ltd. (Shanghai, China). Carveol was obtained from Alfa Aesar (Shanghai, China) Chemical Co. Ltd. (Shanghai, China). The reagents (i.e., NaCl, glucose, FeSO_4_·7H_2_O, ethanol) were purchased from Sinopharm Chemical Reagent Co., Ltd., Shanghai, China.

### 3.2. Screening of Limonene Resistant Microorganisms

The eleven distinct samples were chosen to isolate and screen microorganisms. Samples were collected from soil of citrus processing plant (S1), soil of citrus fruits (S2), soil of Jianghan Oilfield (Qianjiang, Hubei, China) (S3), peel of fresh and rotten citrus fruits. The used citrus fruits included Gannan navel orange (*Citrus sinensis* Osbeck, fresh-S4, rotten-S5), Anyue lemon (*Citrus limon* (L.) Burm. f., fresh-S6, rotten-S7), Guanxi pomelo (*Citrus grandis* (L.) Osbeck.cv.guanxi miyou, fresh-S8, rotten-S9), and Satsuma mandarin (*Citrus unshiu* Marc., fresh-S10, rotten-S11). The collected samples were stored in aseptic packing.

Screening of limonene-resistant microorganisms was performed as described previously [17,18]. Firstly, each sample (10 g) collected above was transferred to 90 mL of sterile distilled water and incubated for 20 min at 30 °C and 150 rpm. Subsequently, 5 mL inoculums and 50 μL (0.1%, *v/v*) limonene were transferred to 50 mL yeast and malt extract (YM) medium (glucose 10 g/L, peptone 5 g/L, malt extract 3 g/L, yeast extract 3 g/L). After 2 or 7 days of cultivation, a 100 μL sample of the culture broth was spread onto the YMA (YM medium with 2.0% (*w/v*) agar) medium plates. The plates were incubated at 30 °C until colonies grew completely, limited to 7 days. After each different colony appeared, the microorganisms were transferred to YM medium and grown at 30 °C using constant agitation (150 rpm).

Secondly, each isolated strain (100 μL) and 100 μL (2%, *v/v*) of limonene were added to 5 mL of YM medium and incubated for 48 h at 30 °C and 150 rpm. Afterwards, a 100 μL sample of the culture broth was spread onto the YMA plates. The culture growth was evaluated after 48 h at 30 °C. The strains that presented a satisfactory growth were considered resistant to 2% limonene [18].

Finally, the isolated colonies were evaluated whether they can utilize limonene as the sole carbon source. Pre-cultures (1 mL) of the isolated colonies was inoculated into 100 mL of YM medium and incubated for 12 h at 30 °C and 150 rpm. After incubation, cells were harvested by centrifugation and washed with saline water. Cells were re-suspended in M9 medium (Na_2_HPO_4_ 6 g/L, NaCl 0.5 g/L, KH_2_PO_4_ 3 g/L, NH_4_Cl 1 g/L, 1 mol/L MgSO_4_ 2 mL). Optical density at 600 nm was adjusted to 0.2. Limonene (1%, *v/v*) was added and the cultures were incubated for 6 d at 30 °C and 150 rpm. The optical density was measured at 600 nm every 12 h to evaluate the cell growth [17]. The blank experiment without inoculation was also carried out. For fungi, the culture was harvested by vacuum filtration. The mycelia were washed with saline water and then re-suspended into the same volume of M9 medium. Limonene (1%, *v/v*) was added and cultures were incubated for 6 d. The biomass was filtered and quantified by dry weight to verify the growth of the microorganisms [14].

### 3.3. Identification of Limonene Resistant Microorganisms

The potential isolates were identified by 16S rDNA or ITS sequencing and submitted to GenBank agreement. Genomic DNA from the isolates was extracted using genomic DNA isolation reagent (Sangon Biotech Co., Ltd., Shanghai, China). Amplification of the 16S rDNA and ITS gene was performed using Taq PCR MasterMix with the universal primers 21F/1492R and ITS1/ITS4 for bacteria and fungi, respectively (27F, AGAGTTTGATCCTGGCTCAG; 1492R, TACGGTTACCTTGTTACGACTT; ITS1, TCCGTAGGTGAACCTGCGG; ITS4, TCCTCCGCTTATTGATATGC). Then, PCR products were sequenced and compared using the basic local alignment search tool (BLAST) program of National Centre for Biotechnology Information (NCBI). The sequences of isolated strains were aligned with the previously reported sequences of different closely related species using ClustalW multiple sequence alignment. Moreover, phylogenetic tree was built using the Neighbour-Joining method through MEGA 7.0. The robustness of the tree was estimated by bootstrap analysis based on 1000 replications.

### 3.4. Limonene Biotransformation

Biotransformation experiments were conducted in a 250 mL conical flasks containing 100 mL of medium. Pre-cultures (1 mL) of fungi was inoculated into 100 mL of YM medium and incubated for 36 h at 30 °C and 150 rpm. Pre-cultures of bacteria was transferred into Luria-Bertani (LB) medium (peptone 10 g/L, yeast extract 5 g/L, NaCl 10 g/L) for 4 h, to reach an optical density at 600 nm close to 1.2. Subsequently, limonene (840 mg/L) was added and biotransformation process was monitored at 24 h, 48 h and 72 h by solid-phase microextraction (SPME)/gas chromatography-mass spectrometry (GC-MS) using the cyclohexanone as an internal standard [52]. Blank assays without limonene and without microorganism were carried out.

### 3.5. The Optimization of Trans-Dihydrocarvone Production by Klebsiella sp. O852 Using Single Factor Design

Various fermentation conditions, including metal ion, biotransformation time, cultivation time, substrate concentration, co-solvent, temperature and agitation, were optimized for *trans*-dihydrocarvone production, using the classical approach of varying only one variable per test. Optimized conditions were then used sequentially in subsequent fermentations. The detailed methods as follows.

#### 3.5.1. Growth Curve of Klebsiella sp. O852

Pre-cultures (1 mL) was inoculated into 100 mL of LB medium and incubated at 30 °C and 150 rpm. The OD_600_ of *Klebsiella* sp. O852 was determined every 4 h using UV-1700 spectrophotometer.

#### 3.5.2. Effect of Metalions on the Production of Trans-Dihydrocarvone by *Klebsiella* sp. O852

Pre-cultures (1 mL) of *Klebsiella* sp. O852 was transferred into 100 mL of LB medium supplemented with different sources of metal ions (Zn^2+^, Ca^2+^, Mn^2+^, Cu^2+^, Fe^2+^, Mg^2+^, 0.4 g/L) and incubated for 4 h at 30 °C and 150 rpm. The biotransformation process was monitored for 24 h after adding 840 mg/L limonene by SPME/GC-MS. Blank assay without metal ion was carried out. Conversion ratio was calculated using the following equation.

(1) $$\mathrm{Conversion}\;\mathrm{ratio}\;\left(\%\right)=\frac{trans-dihydrocarvone\;concentration}{limonene\;concentration}\times 100\%$$

#### 3.5.3. Effect of Biotransformation Conditions on the Production of Trans-Dihydrocarvone by *Klebsiella* sp. O852

Pre-cultures (1 mL) was transferred into 100 mL of LB-M medium (LB medium with 0.4 g/L Fe^2+^) and incubated for different cultivation time (4 h, 8 h, 12 h, 16 h, 20 h and 24 h) at 30 °C and 150 rpm. The process was monitored for different biotransformation time (12 h, 24 h, 36 h and 48 h) after adding different concentrations of limonene (168 mg/L, 420 mg/L, 840 mg/L, 1680 mg/L and 2520 mg/L).

#### 3.5.4. Effect of Co-Solvent on the Production of Trans-Dihydrocarvone by *Klebsiella* sp. O852

Pre-cultures (1 mL) was transferred into 100 mL of LB-M medium and incubated for 4 h at 30 °C and 150 rpm. The process was monitored for 36 h after adding 1680 mg/L limonene dissolved in co-solvent. Limonene was used as a 20% (*v/v*) solution in the different types of organic solvents (ethanol, glycerol, glycol, ethyl acetate, acetone, methanol and dimethyl sulfoxide), leading to a final co-solvent concentration of 0.8% (*v/v*). Blank assay without co-solvent was carried out.

Besides, optimized co-solvent was selected and its concentration was adjusted 0.2%, 0.6%, 0.8%, 1.2%, 1.6% and 2.0% (*v/v*), respectively. Biotransformation process was monitored after adding 1680 mg/L limonene/co-solvent.

#### 3.5.5. Effect of Culture Conditions on the Production of Trans-Dihydrocarvone by *Klebsiella* sp. O852

Pre-cultures was transferred into LB-M medium for 4 h at different temperatures (24 °C, 28 °C, 30 °C, 36 °C and 40 °C) and different agitation speeds (100 rpm, 150 rpm, 200 rpm, 250 rpm and 300 rpm). The process was monitored for 36 h after adding 1680 mg/L limonene/ethanol (final ethanol concentration of 0.8% (*v/v*)).

### 3.6. Statistical Analysis

Data were expressed as mean ± standard deviations (SD). Statistical analysis was determined using the Mann–Whitney test (GraphPad Software, Inc., San Diego, CA, USA). The level of significance was established at *p* < 0.05 and *p* < 0.01. In addition, statistical analysis of Table 1 was performed by one-way ANOVA in SPSS 16 (SPSS Inc., Chicago, IL, USA).

## 4. Conclusions

In this study, a new strain O852 was isolated from Gannan navel orange, which was proved to be capable of converting limonene to *trans*-dihydrocarvone. The isolate was identified as *Klebsiella* sp. based on the 16S rDNA gene sequence comparison, and it was named as *Klebsiella* sp. O852. Subsequently, the optimization of this strain to produce *trans*-dihydrocarvone was studied using single factor experiment. The results showed that *Klebsiella* sp. O852 was cultured in LB-M medium for 4 h (logarithmic growth phase) at cultivation conditions of 36 °C and 150 rpm. The concentration of substrate limonene was 1680 mg/L, and the biotransformation time was 36 h after adding substrate. Ethanol was used as a co-solvent with a final concentration of 0.8% (*v/v*). The maximum yield of *trans*-dihydrocarvone was 1 058 mg/L and the conversion ratio was 63%. The content of *trans*-dihydrocarvone increased 16 times after optimization. The wild-type strain *Klebsiella* sp. O852 might serve as a candidate for the biotransformation of limonene to *trans*-dihydrocarvone as an efficient biocatalyst. The focus of further work will be on the molecular basis of reaction and enzymatic modulations.

## Figures and Tables

**Table 1:** Concentration (mg/L) of limonene biotransformation products obtained at different periods (24 h, 48 h, 72 h).

Product	RI (Measurement)	Content (mg/L)						ID
		*Klebsiella* sp. O852			*Debaryomyces* sp. O97			
		24 h	48 h	72 h	24 h	48 h	72 h	
γ-terpinene	1097	2.36 ± 0.15 ^a^	4.25 ± 1.3 ^ab^	3.26 ± 0.8 ^b^	4.29 ± 0.85 ^b^	5.28 ± 0.28 ^a^	4.78 ± 1.02 ^b^	B
1,2-limonene epoxide	1171	15.41 ± 2.67 ^b^	7.3 ± 1.92 ^b^	11.12 ± 1.47 ^c^	11.56 ± 2.28 ^c^	4.68 ± 1.1 ^a^	1.5 ± 0.52 ^a^	B
*trans*-dihydrocarvone	1235	62.86 ± 5.95 ^c^	45.26 ± 3.83 ^c^	16.96 ± 1.98 ^d^	n.d.	n.d.	n.d.	A
p-menth-1-en-9-al	1248	2.37 ± 0.26 ^a^	1.74 ± 0.23 ^a^	1.39 ± 0.19 ^ab^	n.d.	n.d.	n.d.	B
*trans*-carveol	1263	n.d.	n.d.	n.d.	n.d.	15.01 ± 3.28 ^a^	12.13 ± 2.06 ^c^	A
carvone	1283	n.d.	8.93 ± 0.12 ^b^	n.d.	n.d.	64.03 ± 22.09 ^b^	n.d.	A
perillyl alcohol	1333	n.d.	n.d.	n.d.	1.43 ± 0.47 ^a^	1.82 ± 0.11 ^a^	0.96 ± 0.02 ^a^	A

n.d., not detected. ID, identification: A, comparison of mass spectra and retention index with authentic standards; and B, comparison of mass spectra with the MS library of Wiley7.0 and NIST 0.5. RI, retention index on a HP-5 column, was calculated using a mixture of n-paraffins (C7–C30) as standards. Results were given as the mean ± standard deviation (*n* = 3). ^a,^ ^b,^ ^c,^ ^d.^ Statistical analysis ANOVA (*n* = 3) at 95% confidence level with same letters indicating no significant difference.

## Data Availability

The data presented in this study are available on request from the corresponding author.

## Sample Availability

Samples of the compounds are available from the authors.
